# Awareness and perception of pharmacy researchers about conflict of interest: A study from the MENA region

**DOI:** 10.7324/japs.2022.120815

**Published:** 2022-08-04

**Authors:** Hadeia Mashaqhbeh, Karem H. Alzoubi, Omar F. Khabour, Rana Abu-Farha

**Affiliations:** 1 Department of Pharmaceutical Technology, Jordan University of Science and Technology, Irbid 22110, Jordan; 2 Department of Pharmacy Practice and Pharmacotherapeutics, University of Sharjah, Sharjah, UAE.; 3 Department of Clinical Pharmacy, Jordan University of Science and Technology, Irbid 22110, Jordan; 4 Department of Medical Laboratory Sciences, Jordan University of Science and Technology, Irbid 22110, Jordan; 5 Department of Clinical Pharmacy and Therapeutics, Faculty of Pharmacy, Applied Science Private University, Amman 11931, Jordan

**Keywords:** Pharmacy researchers, attitude, conflict of interest, MENA

## Abstract

The literature has shown some bias in research about the effectiveness and efficacy of medications due to the influence of the pharmaceutical industry. Thus, researchers’ understanding of conflict of interest (COI) is substantial and may influence the quality of scientific research. Therefore, in this study, we aimed to assess pharmacy researchers’ awareness and perception about COI in the Middle East and North Africa (MENA) region and the factors affecting their disclosure of COI. In this cross-sectional study, a convenience sample of pharmacy researchers from the MENA region was surveyed for their awareness and perception about the principles of COI. The questionnaire was divided into three main sections. The first section assesses the demographic information of the study participants; the second section assesses researchers’ awareness and practices in dealing with COI; and, finally, the third section assesses participants’ perception about COI. The questionnaire was uploaded on the Google Forms electronic platform, and it was distributed to the potential study participants via their professional emails. Finally, logistic analysis was used to evaluate predictors affecting COI disclosure by the study participants. A total of 271 pharmacy researchers participated in this study. Researchers were distributed based on their academic rank as follows: 63 professors (23.3%), 59 associate professors (21.8%), 97 assistant professors (35.8%), and 52 lecturers (19.2%). The majority of researchers indicated that they were familiar with the concept of COI (88.9%), and they agreed that the existence of COI should be disclosed by all authors (84.5%). About 33.2% of the study participants did not know if their institution had a policy for COI. About half of the participants (48.70%) believed that disclosure is the only way for the management of COI. However, only 41.7% of them (*n* = 113) indicated that they had disclosed COI in their published research. Finally, participants who have a collaboration with the industry showed a significantly lower tendency to disclose their COI than those who do not have any partnership with the industry (OR = 0.471, *p* = 0.017). The majority of the pharmacy researchers from the MENA region are familiar with the principles of COI, but a lower percentage of them disclose their COI in their published research. Efforts should be made to educate researchers about the importance of disclosing COI, which might reduce the potential of bias in their published research.

## INTRODUCTION

Conflict of interest (COI) is defined as a “circumstance that creates a risk where professional judgments or actions regarding a primary interest will be unduly influenced by a secondary interest” (Field and Lo, 2009). The COI scope is not only financial; it can also include other forms, such as intellectual, professional, and institutional (Arend, 2017; Rohwer *et al*., 2017; Sharma, 2020). These could also have different ethical implications and should be reported in publications (Barber, 2020; Grinnell, 2014). For example, researchers with COI are at increased risk of irrelevant findings, decreased objectivity, impaired scientific judgment, and any other considerations that influence their professional decision-making (Cherla *et al*., 2019; Sharma, 2020). The consequences of COI could range from direct data fabrication or falsification to influence on the study design, explanation of data, and/or choice of studies or literature to make arguments (Bero, 2017). Other more subtle effects include reduced willingness to share data and lower concerns about the rights of research subjects and general health of the public, but rather ensuring financial profits of sponsors (Elliott, 2008).

The magnitude of COI could be determined by the value of the second interest and its undue influence on the decision-making and the predicted harm or risk that might result from this effect (Acquavella, 2019; Cienfuegos and Perez-Cuadrado Martinez, 2019; Field and Lo, 2009; Galandiuk, 2019). As stated by the World Association of Medical Editors, “COI in medical publishing impacts everyone with a stake in research integrity including journals, research and academic institutions, funding agencies, the popular media, and the public” (Ferris and Fletcher, 2012). The policies of COI usually focus on financial benefits. These policies concern management and prevention of COI rather than punishment, as they do not assume that financial benefits or any secondary interest could influence the professional’s judgment (Cherla *et al*., 2019; Samsa and Solomon, 2019). However, evidence exists showing that COIs influence professional judgment (Elliott, 2008). Thus, the most common strategy to manage and address COI is the disclosure of conflict (Boyd and Bero, 2007; Glaser and Bero, 2005). To improve the management of COI, an enforcement system called “Open Payments” was adopted in the United States (Slentz *et al*., 2019; Tisherman *et al*., 2021). The system is an open database that contains information on payments to academic researchers made by the industry (Chabner and Bates, 2018). Additionally, scientific reviewers who can assess the rigor and transparency of the research in fair and efficient ways are another layer to ensuring proper management of COI (Samsa and Solomon, 2019).

Institutions and universities usually establish or adopt policies and roles for reporting COI. Journals also request authors to disclose any of their relevant financial relations or any possible COIs (Hams *et al*., 2017; Resnik *et al*., 2016). Thus, researchers are required, by the journal or by their institutions, to report all the financial support or even material support during the conduct of the research (Fontanarosa and Bauchner, 2017; Thornton, 2017). The literature has shown some bias in research reports on the effectiveness and efficacy of medications due to the influence of the pharmaceutical industry (Garattini *et al*., 2020; Lexchin, 2012; Saito *et al*., 2019; Wranik *et al*., 2019). In addition, even in developed countries, not all medical schools and teaching hospitals have COI policies, and those that have one demonstrated differences in its content (Fabbri *et al*., 2021; Guy-Coichard *et al*., 2019). Studies from the Middle East and North Africa (MENA), regarding COI are scarce (Al-Zyoud *et al*., 2019; Nakkash, 2018; Nasr *et al*., 2020; Sleem *et al*., 2010). In addition, not all institutional review boards in MENA have established policies to manage conflicts of interest ( Al-Zyoud *et al*., 2019; Sleem *et al*., 2010). Furthermore, about 40% of the researchers from low- and middle-income countries reported not having declared a COI in past researches (Rohwer *et al*., 2017). A study that included researchers who had participated in at least 10 clinical trials showed considerable variability in researchers’ understanding of the components of the COI and when such components should be reported (Østengaard *et al*., 2020). As pharmaceutical researchers are of particular interest, they often have partnerships with the pharmaceutical industry, which can increase their exposure to the impact of COI. In addition, the knowledge and practices of pharmacy researchers from the MENA region on COI have not yet been examined. Therefore, in this study, we aimed to assess pharmacy researchers’ awareness and perception about COI in the MENA region and the factors affecting the disclosure of their COI.

## METHODS

### Study design and study participants

This is a survey-based cross-sectional study that was conducted to evaluate pharmacy researchers’ awareness and perception about COI and to evaluate factors affecting their disclosure of COI. A convenience sample of pharmacy researchers working in academic institutions from the MENA region was targeted in this study.

### Questionnaire development and data collection

The draft version of the questionnaire was developed by the research team following a comprehensive literature review of relevant investigations (Schwartz *et al*., 2007; Schetky, 2008). The questionnaire was tested for face and content validity by two experts in research ethics to ensure the clarity and the comprehensibility of the questions. Expert inputs were taken into consideration, and the questionnaire was modified consequently. After that, a pilot testing on 10 pharmacy academics was performed, where academics were asked to deliver their comments about the clarity and understandability of the questions included. Data collected during this pilot testing were not included in the final analysis of the results.

The questionnaire was divided into three main sections. The first section assesses the demographic information of the study participants, including gender, nationality, current academic rank, and the number of published papers; the second section assesses researchers’ awareness and practices in dealing with COI; and, finally, the third section assesses participants’ perception about COI. This section was evaluated using a 3-point Likert scale where “1 = disagree, 2 = neutral, and 3 = agree.”

The questionnaire was then uploaded on the Google Forms electronic platform, and it was distributed to the potential study participants via their professional emails. The first page of the questionnaire contains details about the study objective and voluntariness of participation, followed by an electronic consent where researchers were given the choice to voluntarily agree to participate or decline participation. The participants were informed that they did not have to disclose their identity and that data would be kept on the computer of the principal investigator using password-protected files.

### Sample size calculation

The standard formula *n* = *P* × (1−*P*) × *z*^2^/*d*^2^ was used to calculate a minimal sample size, which was determined based on the most conservative proportion (*P* = 50%) of researchers to disclose their COI, using 10% desired precision, and confidence levels of 95%. Therefore, 96 pharmacy researchers were considered the minimum required sample size needed for this study.

### Ethical consideration

Before study conduction, approval was obtained from the Institutional Review Board (IRB) of Jordan University of Science and Technology (JUST) (Reference No. 72/124/2019). The guidelines issued by the World Medical Association (2013), Declaration of Helsinki, was followed in the study. Participants were informed about the voluntariness of their participation. As per IRB permission, electronic informed consent was obtained from each participant, where the questionnaire would not open unless participants provided their approval to participate in this study.

### Statistical analysis

The Statistical Package for the Social Sciences (SPSS^®^) version 22 was used for data management and analysis. Categorical variables were expressed as frequency (percentages). Logistic regression analysis was used to evaluate the relationship between several predictors (including gender, academic rank, number of published papers, familiarity with the COI concept, and having a partnership with industry) and COI disclosure by the study participants. For this purpose, the number of published papers was divided using the 15-publication benchmark as this represents the average minimum number of publications needed to fulfill the requirement of full professor rank in universities in MENA. Univariate logistic regression was performed initially, and any variable that showed a *p* value< 0.25 was considered eligible for entry in the multiple logistic regression analysis. Multicollinearity between variables was checked using the Pearson correlation coefficient, where *r* < 0.09 indicates the absence of multicollinearity between the independent variables. Following multiple logistic regression, any variable with *P* <0.05 was considered a statistically significant predictor.

## RESULTS

As presented in Table 1, 271 academic pharmacy researchers from the MENA region participated in this study. 58% of the study sample were male (*n* = 157), while females 42.1% of the respondents were female (*n* = 114). Researchers were from Jordan (*n* = 80, 29.5%), Egypt (*n* = 72, 26.6%), Saudi Arabia (*n* = 26, 9.6%), Iraq (*n* = 20, 7.4%), and other countries (*n* = 73, 26.9%). Distribution of participants per academic rank was professors (*n* = 63, 23.3%), associate professors (*n* = 59, 21.8%), assistant professors (*n* = 97, 35.8%), and lecturers 19.2%). Slightly less than half of the respondents (*n* = 122, 45.7%) published more than 15 research papers, while around 4.9% of them (*n* = 13) published more than 100 research papers. The remaining respondents published between 16 and 30 publications (*n* = 63, 23.6%) or between 31 and 100 publications (*n* = 69, 25.8%). Moreover, only 22.9% of the researchers (*n* = 62) reported that they have/had collaboration/funding with the industry, while the remaining 77.1% (*n* = 209) reported that they had no collaboration/funding with the industry. For more details about the sociodemographic and academic information, refer to Table 1.

In this study, 88.9% of the researchers (*n* = 241) indicated that they were familiar with the COI concept, while only 11.1% of them (*n* = 30) were not familiar with the concept, as presented in Table 2. More than one-third (*n* = 108, 39.9%) of them knew that their institution had a policy for COI. Most of the participants (*n* = 219, 80.8%) agreed that disclosing COI is among the ways to manage COI, while others (*n* = 48, 17.7%) believed that avoiding the presence of COI is the best way to manage COI. Lastly, only 41.7% of the study participants (*n* = 113) indicated that they had disclosed COI in their published research.

Participants’ perception toward conflict of interest was evaluated (Table 3). The majority of participants agreed that COI should be disclosed by all authors (*n* = 229, 84.5%), as well as by reviewers and editors (*n*= 196, 72.3%). Most of the participants (*n* = 225, 94.1%) agreed that any financial or material support should be reported in the acknowledgment section of the published articles. Additionally, about half of the researchers (n= 131, 48.3%) believed that disclosure is the only way for COI management. Finally, the majority of participants (n= 215, 79.3%) agreed that COI could influence the integrity and the quality of research.

Finally, logistic regression analysis showed that participants who collaborated with the industry showed a significantly lower tendency to disclose their COI than those who did not have any collaboration with the industry (OR = 0.471, *p* = 0.017) (Table 4), while other variables including gender, academic rank, number of published papers, and familiarity with the COI concept showed no significant association with the tendency of respondents to disclose their COI (*p* > 0.05). Moreover, the logistic regression full model was able to predict more accurately than the null model [χ^2^ (df = 3) = 10.549 at *p* = 0.014].

## DISCUSSION

Limited studies on COI have been conducted in the MENA region (Al-Zyoud *et al*., 2019; Heidari *et al*., 2012; Rababa’h *et al*., 2020). In this study, the awareness and practices of pharmacy researchers from the MENA region were investigated. The majority of academic researchers of various pharmaceutical disciplines indicated that they were familiar with the concept of COI and had a positive attitude toward the disclosure of COI. Most of the participants agreed that COI should be disclosed by all authors. This is consistent with a previous study conducted among researchers from several low- and middle-income countries, which showed that most of the health researchers agreed that financial conflict should be disclosed in the research project (Rohwer *et al*., 2017). In another study from the USA that examined researchers’ awareness and attitudes toward COI, researchers suggested the need for extra efforts to increase understanding of the relevance of COI policies for all academics included in the study (Lipton *et al*., 2004). Similarly, researchers from China indicated the need to enhance knowledge about COI principles among academics (Yang *et al*., 2021).

In this study, most of the participants agreed that COI should be disclosed by reviewers and editors and agreed that any financial or material support should be reported in their published articles. This is consistent with the policies and procedures of the majority of peer-reviewed journals and international publishers (Breimer *et al*., 2018; El Moheb *et al*., 2021). Moreover, most researchers believed that COI disclosure could impact the integrity/quality of research. It is notable, however, that previous studies have shown that in some instances disclosures of COIs not only fail to prevent the problems of COIs but may make those problems worse (Cain *et al*., 2010). Some researchers might avoid research that includes COI because they believe that COI disclosure is not enough and the quality of research could be influenced even if COI was declared (Rohwer *et al*., 2017). Academic institutional policies usually address COI disclosure, control, and removal via denudation or recusal (Elliott, 2008). In the majority of universities in Jordan, COI policies were reported to be inadequate (Al-Zyoud *et al*., 2019). However, it was previously argued that such strategies could fall short in the face of the wide range of influences of COI on researchers (Cain *et al*., 2010; Elliott, 2008). Thus, alternative strategies should be considered such as encouraging the conduction of more independent research to ensure scientific reliability and truthfulness (Elliott, 2008).

The unfortunate scenario is where the researcher does not disclose COI. In a study from lower-middle-income countries, 43% of researchers reported that they knew another researcher at their institution who did not disclose financial COI (Rohwer *et al*., 2017). In developed countries, considerable variability was found in clinical studies on researchers’ understanding of components of the COI and when such components should be reported (Østengaard *et al*., 2020). COI (including nonfinancial) information was estimated to be underreported in medical journals (El Moheb *et al*., 2021; Faggion *et al*., 2020). In a study from MENA, COI disclosure was required by about 45% of the Iranian journals (Heidari *et al*., 2012). In a study from Spain, undisclosed COI was reported to be highly prevalent for authors in major journals (Tisherman *et al*., 2020). COI financial reports for drug trial authors in meta-analyses and systematic reviews published in high-impact journals have been shown to be suboptimal (Benea *et al*., 2020). Comparably, the participants of the current study had a positive attitude toward the declaration of COI; however, only 41.7% of respondents reported COI. The reasons behind such a discrepancy between attitude and actual practice of COI disclosure are unknown. Probably, a comprehensive qualitative study could give more in-depth insight into this issue. In a study that was conducted in China, most of the underreporting was attributed to the lack of knowledge about COI, followed by concern that a COI would lead to rejection of the manuscript by the journals (Yang *et al*., 2021). Efforts should be made by research and academic institutions in the MENA region to train the researchers on COI and the importance of financial and nonfinancial COI disclosure.

The present results showed that pharmacy researchers collaborating with the industry reported a significantly lower tendency to declare COI than those without any industry partnership. Partnerships between academic researchers and the industry could be associated with a kind of bias in the results or the design of the research project. For example, a study showed that funding of drug and device studies by the industry leads to more favorable findings and conclusions than funding from sources other than the industry (Lundh *et al*., 2017). Some have argued that the main reason for positive results in industry-funded research is that the industry typically sponsors studies in which results are likely to be positive (Barbieri and Drummond, 2001). A previous study conducted on plastic surgeons showed discordance between researchers self-reporting in scientific journals and the government-mandated reporting of COI by the industry (Lopez *et al*., 2018). In a study from developing countries, researchers believed that, even with funding declaration, partnerships with commercial companies could affect researchers at some level in their research (Rohwer *et al*., 2017). This could explain why researchers with a partnership with the industry tend not to disclose COI. Moreover, as the collaboration with the industry could restrict researchers from controlling publication decisions and may affect study design, researchers should be aware of their responsibilities as authors to guarantee study integrity (Hirsch, 2009). Thus, relationships with the industry provide opportunities for innovative research, but not declaring COI may undermine confidence in academic integrity (Ross *et al*., 2020).

One way to improve COI management and reporting is to include the concepts of COI as an integral part of medical students’ undergraduate curriculum and education ( Andresen *et al*., 2017; Bechoux *et al*., 2021; Deis *et al*., 2020), thus, avoiding COI cases among pharmacists who are likely to become future researchers and experts working with the drug industry. A previous study from Jordan showed that the majority of graduating-year pharmacy students were unaware of COI, which was related to the limited coverage of COI concepts in their pharmacy curriculum (Ababneh *et al*., 2020). In a study that was conducted in France, about 85% of the medical students reported feeling inadequately educated about COI (Deis *et al*., 2020). Thus, it is evident that university medical curricula in some countries lack key concepts related to COI, disclosure of financial interests, and the ethical obligation to declare COI.

Finally, it is important to highlight that there were some limitations that must be pointed out in this study. First, the study data were collected via a self-administered questionnaire which might have generated a risk of bias since the participants would possibly display socially desirable answers. In addition, the sample size needed was calculated based on a formula for prevalence study. However, logistic regression analysis was also conducted which may need a larger sample size. Furthermore, the response rate was not calculated in this study because of the inability to determine the number of academics who received the email within the contacted institutions. The study should be expanded to include researchers from other fields. Finally, the actual practices to manage COI in research should be addressed in future investigations.

## CONCLUSION

Pharmaceutical researchers indicated that they were generally familiar with the concept of COI and believed that an existing conflict should be disclosed. This study reveals that the awareness of pharmaceutical academic researchers in the MENA region about the strategies for COI management and their disclosure practice need to be improved. Finally, industry collaborators showed a lower tendency to disclose their conflict of interest than noncollaborators. Thus, efforts should be made to educate researchers about the importance of disclosing their COI which might reduce the potential of bias in their published research. Future studies should explore in-depth the reasons behind not reporting COI by some researchers. In addition, evaluation of the effectiveness of the currently implemented strategies to manage and mentor COI in the MENA region is needed.

## Figures and Tables

**Table 1.** Sociodemographic and academic characteristics of the study participants (*n* = 271).

Variable	*n* (%)

Gender	
• Female	114 (42.1)
• Male	157 (57.9)
Country	
• Jordan	80 (29.5)
• Egypt	72 (26.6)
• Saudi Arabia	26 (9.6)
• Iraq	20 (7.4)
• Other countries	73 (26.9)
Academic rank	
• Professor	63 (23.3)
• Associate professor	59 (21.8)
• Assistant professor	97 (35.8)
• MSc holder	52 (19.2)
Number of published papers	
• ≤15	122 (45.7)
• 16–30	63 (23.6)
• 31–100	69 (25.8)
• More than 100	13 (4.9)
Do you have research with a partnership or funding from industry?	
• No	209 (77.1)
• Yes	62 (22.9)

**Table 2.** Participants’ awareness and practice in dealing with conflict of interest (*n* = 271).

Question	*n* (%)

Are you familiar with the concept of conflict of interest?	
• Yes	241 (88.9)
• No	30 (11.1)
Does your institution have a policy for conflict of interest management?	
• Yes	108 (39.9)
• No	73 (26.9)
• I don’t know	90 (33.2)
As a researcher the best way to manage conflict of interest is to:	
• Disclose the conflict of interest	219 (80.8)
• Avoid the presence of the conflict of interest	48 (17.7)
• Missing data	4 (1.5)
Do you disclose conflict of interest in your published research?	
• Yes	113 (41.7)
• No	158 (58.3)

**Table 3.** Participants’ perceptions toward conflict of interest (*n* = 271).

Statements	Percent agreed *n* (%)

The existence of a conflict of interest should be disclosed by all authors.	229 (84.5)
The existence of a conflict of interest should be disclosed by reviewer and editors.	196 (72.3)
Any financial or material support should be reported in the acknowledgment section	255 (94.1)
Disclosure is the only way for conflict of interest management.	131 (48.3)
The existence of conflict of interest could impact the integrity/ quality of research.	215 (79.3)

**Table 4.** Assessment of the factors associated with conflict of interest disclosure (*n* = 271).

Parameter	COI disclosure [0: No, 1: Yes]			

	OR	*p* value^#^	OR	*p* value^$^

Gender				
• Female	Reference			
• Male	1.510	0.109^^^	1.650	0.059
Academic rank			—	—
• Full professor	Reference			
• Associate professor	1.058	0.879		
• Assistant professor	1.004	0.990		
• Lecturer (M.Sc. holder)	0.628	0.254		
Amount of published research				
• ≤ 15 publications	Reference		—	—
• > 15 publications	0.807	0.409		
Familiar with COI concept				
• No	Reference			
• Yes	1.995	0.149^^^	2.097	0.120
Have a partnership with industry				
• No	Reference			
• Yes	0.543	0.046^^^	0.471	0.017*

# Using simple logistic regression.

$ Using multiple logistic regression.

^ Eligible for entry in multiple logistic regression.

* Significant at the 0.05 significance level.

## Data Availability

The datasets used and/or analyzed during the current study are available from the corresponding author on reasonable request.
